# Comparison of the canal transportation of ProTaper GOLD, WaveOne GOLD, and TruNatomy in simulated double-curved canals

**DOI:** 10.1186/s12903-021-01854-z

**Published:** 2021-10-16

**Authors:** HyeWon Kim, Su-Jin Jeon, Min-Seock Seo

**Affiliations:** Department of Conservative Dentistry, Wonkang University Daejeon Dental Hospital, 77 Dunsan-Ro, Seo-Gu, Daejeon, 302-120 Republic of Korea

**Keywords:** Root canal preparation, Canal transportation, Preparation time, Simulated canals, ProTaper GOLD, WaveOne GOLD, TruNatomy

## Abstract

**Background:**

In root canal preparations, it is important to maintain the original canal shape. However, it is difficult to accomplish this, especially due to the complex canal anatomy. This study aimed to compare the shaping ability of the ProTaper GOLD, WaveOne GOLD, and newly developed TruNatomy in simulated S-shaped canals.

**Methods:**

The root canals of 60 S-shaped resin blocks were dyed using ink and photographed. The blocks were then randomly divided into three groups: group ProTaper GOLD (n = 20), WaveOne GOLD (n = 20), and TruNatomy (n = 20). The simulated canals were instrumented according to the NiTi file system and photographed again after being dyed with red ink. The pre- and post-preparation images were superimposed, and the amount of resin removed from both the mesial and distal sides of the canal measured up to 9 mm from the apical terminus, with a 1 mm increment. The preparation time was also calculated. A paired t-test was used to determine the degree of deviation at different levels within the groups. To compare the degree of transportation at different levels between the groups, one-way ANOVA and Kruskal–Wallis tests were performed according to the normality.

**Results:**

TruNatomy showed a significant deviation between the mesial and distal sides of the canal only in the coronal area at 6, 7, 8, and 9 mm levels of the canal (*p* < 0.05). When comparing the amount of transportation in the 3 groups at 9 different levels, TruNatomy showed significantly less canal transportation than the other groups at the 3-and 5-mm levels of the canal (*p* < 0.05), while ProTaper GOLD showed the largest amount of transportation in the apical curved area at the 2 and 3 mm levels (*p* < 0.05). TruNatomy removed less resin than other groups in all sections (*p* < 0.05), while ProTaper GOLD removed slightly more resin than WaveOne GOLD; however, there was no significant difference (*p* = 0.043). Shaping time was the least for TruNatomy, followed by the WaveOne GOLD and ProTaper GOLD (*p* < 0.05).

**Conclusions:**

TruNatomy maintained the original apical canal curvature in S-shaped curved canals better than ProTaper GOLD and WaveOne GOLD.

## Background

The main purpose of root canal preparation is to remove infected and necrotic pulp tissues in the canal. Simultaneously, it is also important to maintain the original shape of the root canal and preserve healthy root dentin for the long-term prognosis of teeth. However, the complex root canal anatomy makes shaping difficult, which can lead to insufficient disinfection and create procedural errors such as canal transportation, ledge, zip, and perforation [1, 2].

NiTi rotary file systems have been developed to increase flexibility and reduce iatrogenic errors with special alloys, different cross-sectional designs, cutting edges, and varying taper. Thermal treatment of NiTi alloy is also one of the manufacturing methods to improve the mechanical performance, by adjusting its transition temperature, while controlling the alloy microstructure. That is, the heat-treated NiTi alloy mainly contains R-phase or martensite, which is more flexible, while the conventional NiTi alloy contains austenite [3, 4]. It has been reported that heat-treated NiTi files have significantly increased flexibility and cyclic fatigue resistance compared to conventional NiTi files [5–7].

Gold heat-treated instruments, which are heat processed after the machining of the files, have been used to reduce machining process defects and to modify the crystalline phase structure [4]. ProTaper GOLD (PTG, Dentsply Sirona, Ballaigues, Switzerland) and WaveOne GOLD (WOG, Dentsply Sirona) are representatives of gold heat-treated instruments. PTG files are rotary sequential systems with a convex triangular cross-section and a progressive taper. According to the manufacturer, PTG instruments have the same geometry as that of ProTaper Universal but manufactured with proprietary thermal treatment so that they have greater flexibility and resistance to cyclic fatigue [8, 9]. WOG files are reciprocating single-file systems modified from WaveOne. WOG systems are improved by gold heat treatment and a new parallelogram cross-sectional design with two cutting edges [6, 10].

Recently, TruNatomy (TRN, Dentsply Sirona) file systems have been developed. These instruments are also manufactured using post-manufacturing thermal treatment. They have off-centered cross section with regressive taper, and just two cutting edges with a slim NiTi wire design, having 0.8 mm maximum flute diameter. According to the manufacturer, the combination of file design and heat treatment allows for greater flexibility and efficient shaping while removing only dentin, wherever clinically required [11, 12].

There have been several studies comparing the shaping ability of PTG and WOG with other NiTi file systems in curved canals, showing their superior performance at centering ability [9, 13, 14]. However, no study has evaluated the shaping ability of PTG, WOG, and newly developed TRN NiTi files in simulated canals. Several recent studies have compared the cyclic fatigue of TRN and other NiTi file systems [12, 15], but no studies have compared the shaping ability of TRN. Therefore, the aim of this study was to compare the shaping ability of PTG, WOG, and TRN in S-shaped simulated canals.

## Methods

Sixty S-shaped simulated canals in clear resin blocks (Endo Training Bloc-S; Dentsply Sirona) were prepared. The simulated canals had a taper of 0.02, an apical diameter of 0.15 mm, a length of 16 mm, an apical curvature of 20° (3.5-mm radius), and a coronal curvature of 30° (5-mm radius).

**Fig. 1:**
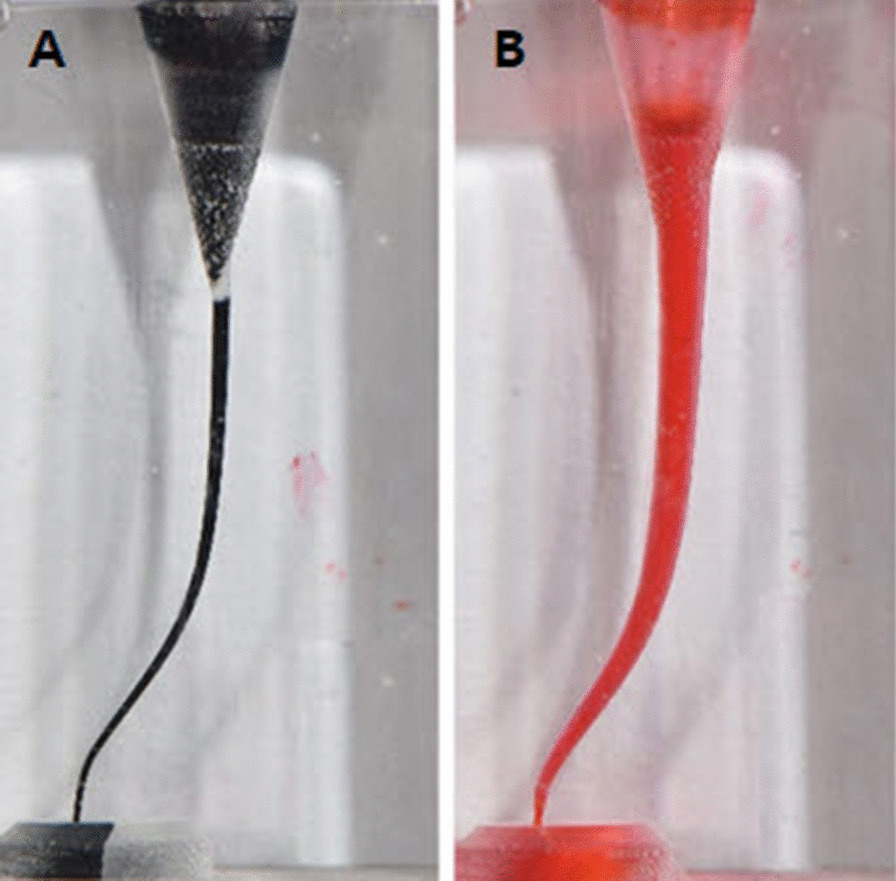
Resin block with S-shaped canal **a** injected black ink before instrumentation and **b** injected red ink after instrumentation

All canals were injected with black ink (Pelikan 400, Pelikan, Hannover, Germany) and photographed using a digital camera (Nikon D5600, Nikon, Tokyo, Japan) in a constant position (Fig. 1a). The resin blocks were positioned on the desk and perpendicular to the floor and the camera was fixed on a tripod parallel to the floor at a distance of 60 cm from the resin blocks. Each canal was rinsed with copious amounts of distilled water before instrumentation. The resin blocks were numbered and randomly sorted into three groups according to the NiTi file systems. After instrumentation, the canals were injected with red ink (Pelikan 400) and photographed again under the same conditions as before (Fig. 1b).

Root canal preparation was performed according to the manufacturer’s recommendations for each system using an X-Smart Plus motor (DentsplyMaillefer,Ballaigues, Switzerland). All procedures were conducted by a second-year resident of the endodontic department, and each NiTi file was used in only one canal. The working length was set at 16 mm, and patency was confirmed using#10 K-file. All instruments were removed from the root canal after three in-and-out motions; the debris on the files cleaned with wet gauze, and the canal irrigated with 2 mL of distilled water each time.

### Group PTG

The PTG S1(18./02), S2(20./04) and F1(20./07), F2(25./08) were used sequentially at 300 rpm and 2 Ncm torque according to the manufacturer’s instructions. After each instrument reached the working length, it was replaced with the next instrument.

### Group WOG

The WOG Primary file (25./07) was used in the “WaveOne Gold” mode of the X-smart Plus motor until the working length was reached. According to the manufacturer’s instructions, the file was gently advanced inwardly with 3 mm amplitude strokes.

### Group TRN

The TRN prime file (26./04) was set at 500 rpm and 1.5 Ncm torque according to the manufacturer’s instructions.

### Assessment of the canal preparation

**Fig. 2:**
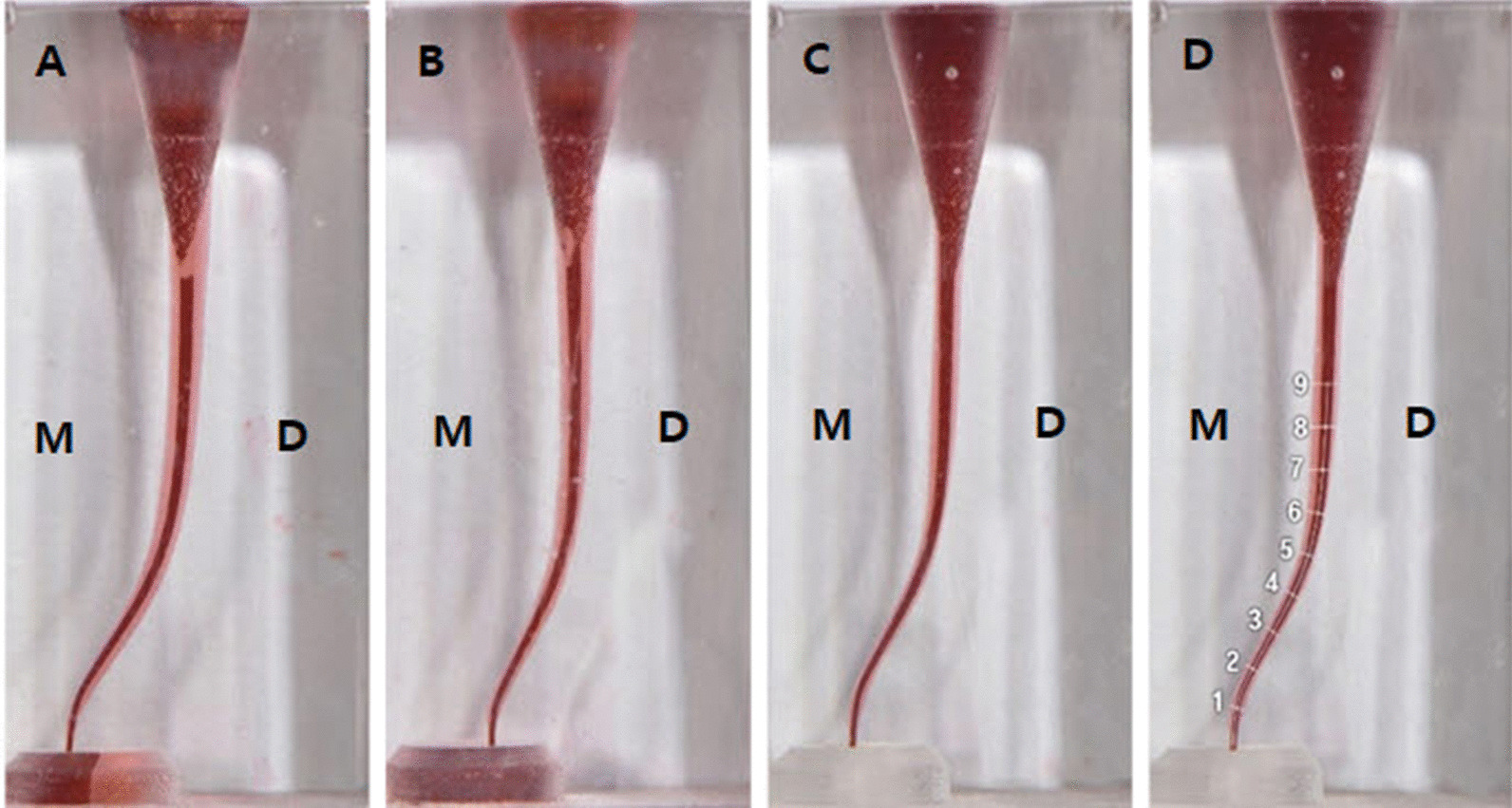
Superimposition images of pre- and post- instrumentation of each group. **a** ProTaper GOLD, **b** WaveOne GOLD, **c** TruNatomy, **d** perpendicular line drawn to the canal central axis at 1 mm intervals from apical foramen. The first measurement point was 1 mm from the apical terminus, and the last point was 9 mm from the apical terminus. M, mesial; D, distal

The pre- and post-preparation images were taken and saved as TIFF. These images were then imported into Photoshop software (Adobe Photoshop CS6, Adobe Systems Inc., San Jose, CA, USA) for superimposition (Fig. 2). The measurement lines perpendicular to the central axis of the canals were drawn at 1 mm intervals from the apical foramen using AutoCAD (AutoCAD LT 2021, Autodesk, San Rafael, CA, USA). The first measurement point was 1 mm from the apical terminus, and the last point was 9 mm from the apical terminus (Fig. 2d). The nine measurement points were divided into three sections, with apical curvature at 1–3 mm levels, coronal curvature at 4–7 mm levels, and straight section at 8–9 mm levels.

The amount of resin removed from the mesial and distal sides of the canals was measured. All measurements were performed under 10X magnification of the superimposed images by the operator of this study and another resident who was blinded to the experimental groups. The amount of transportation was calculated as the absolute value of the difference between the amount of resin removed from the mesial side and that removed from the distal side. The closer the transportation value was to zero, the better the ability to maintain the center.

The preparation time for each canal, including the time taken for active instrumentation, irrigation, cleaning the debris of the files, and changing the instruments, was recorded. Canal shaping was not performed in more than two canals per group in a day so that the operator’s fatigue or skillfulness was excluded.

### Statistical analysis

Statistical analyses were performed using SPSS software (SPSS Statistics 18; SPSS Inc., Chicago, IL, USA). The normality of the data was determined using the Shapiro–Wilk test. In order to determine in which direction the deviation occurred at each level, the values of the resin removed from the mesial and distal sides were compared using a paired t-test. To compare the transportation degree of the intergroup, one-way ANOVA and Kruskal–Wallis tests were performed according to the normality. The post hoc test was performed using Bonferroni’s method. The significance level was set at 5%.

## Results

Table 1 shows the mean amount of resin removed from the mesial and distal sides at 1-mm intervals in each group. When comparing the amount of resin removed from the mesial and distal sides, the PTG and WOG groups showed significant differences at all levels except at the 1 mm and 9 mm levels (*p* < 0.05). On the other hand, in the TRN group, there were significant differences in the amount of resin removed from mesial and distal sides only at 6, 7, 8, and 9 mm levels (*p* < 0.05). Overall, in all three groups, the amount of resin removal was higher on the distal side at 1, 2, 3, and 4 mm, and in the rest of the range, there was more resin removal on the mesial side. When comparing the amount of transportation in the three groups at nine different levels, the TRN group showed less transportation than the other groups at 3-and 5-mm levels (*p* < 0.05). In addition, the PTG group showed the largest amount of transportation at the 2-and 3-mm levels (*p* < 0.05).

**Table 1:** Mean and standard deviation of difference of removed resin materials from mesial and distal side

Level	Instruments											
	ProTaper GOLD				WaveOne GOLD				TruNatomy			
	Mesial	Distal	Difference	*p* value	Mesial	Distal	Difference	*p* value	Mesial	Distal	Difference	*p* value
1	0.103	0.111	0.040 ± 0.028	0.459	0.109	0.123	0.033 ± 0.025	0.108	0.107	0.115	0.029 ± 0.020	0.380
2 a, c	0.085	0.186	0.108 ± 0.049	0.000*	0.115	0.154	0.041 ± 0.029	0.000*	0.101	0.047 ± 0.141	0.351	0.351
3a, b,c	0.105	0.216	0.110 ± 0.045	0.000*	0.056	0.171	0.056 ± 0.032	0.000*	0.019	0.107	0.019 ± 0.014	0.187
4	0.156	0.194	0.442 ± 0.038	0.001*	0.194	0.162	0.441 ± 0.031	0.003*	0.110	0.116	0.231 ± 0.018	0.328
5 a,b, c	0.248	0.163	0.086 ± 0.042	0.000*	0.268	0.131	0.137 ± 0.049	0.000*	0.131	0.114	0.033 ± 0.029	0.083
6 b, c	0.314	0.151	0.163 ± 0.047	0.000*	0.314	0.134	0.180 ± 0.054	0.000*	0.173	0.112	0.067 ± 0.044	0.000*
7	0.319	0.176	0.143 ± 0.075	0.000*	0.275	0.162	0.115 ± 0.062	0.000*	0.224	0.126	0.105 ± 0.055	0.000*
8	0.306	0.216	0.103 ± 0.067	0.000*	0.276	0.229	0.078 ± 0.053	0.013*	0.243	0.151	0.104 ± 0.056	0.000*
9	0.281	0.254	0.069 ± 0.046	0.144	0.256	0.249	0.067 ± 0.068	0.746	0.241	0.171	0.082 ± 0.054	0.000*

Statistically significant differences for transportation (difference): a between PTG and WOG, b between WOG and TRN c between PTG and TRN (*p* < 0.05)

*Statistically significant difference when comparing the amount of removed resin in mesial and distal walls (*p* < 0.05)

**Fig. 3:**
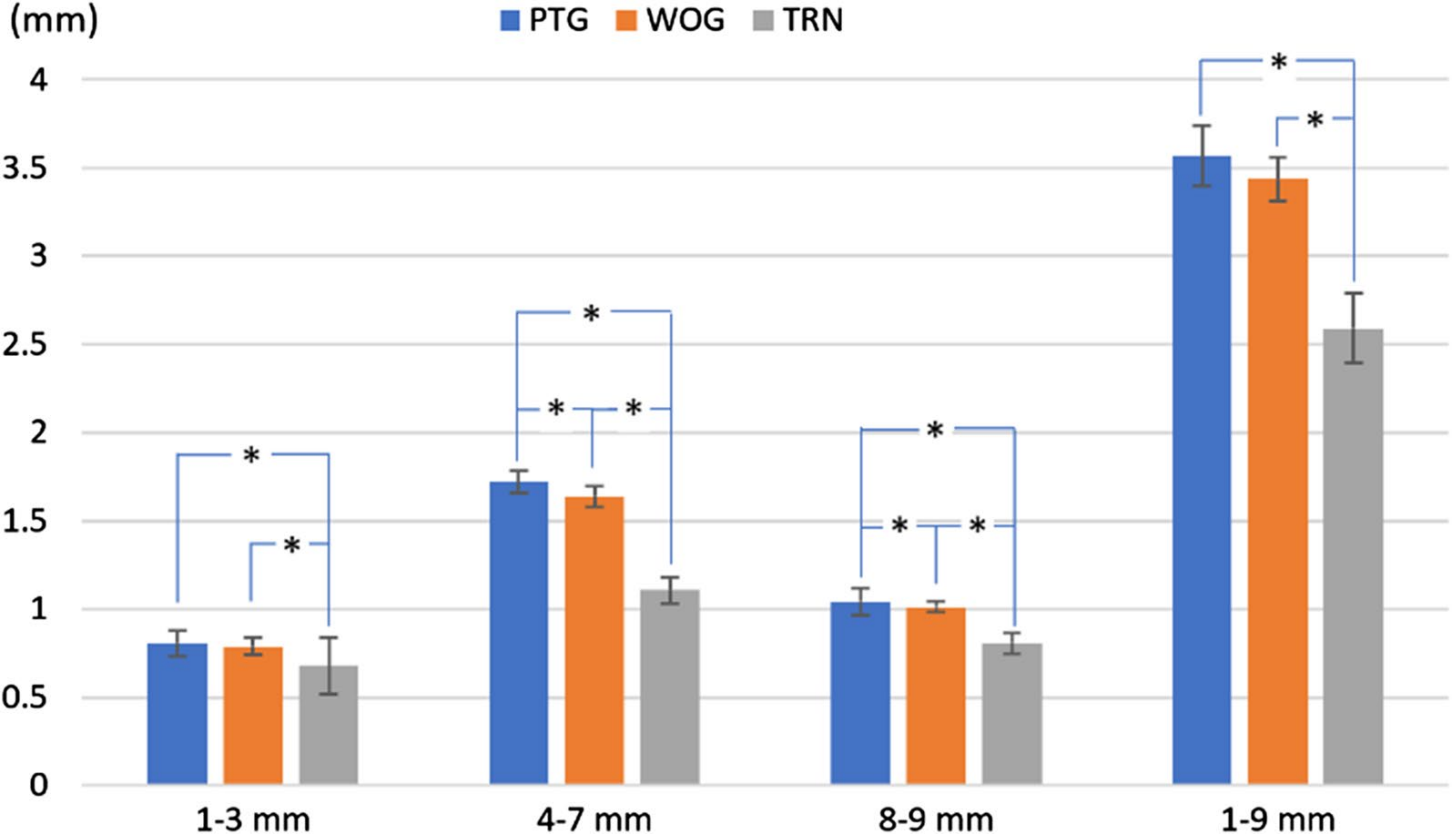
Mean and standardization of the total amount of removed resin for each range

The total amount of resin removed for each section, shown in Fig. 3, was calculated by adding the amount of resin removed from the mesial and distal sides. In the TRN group, less resin was removed than in the other groups, in all the sections (*p* < 0.05). In the entire range, resin removal was slightly more in the PTG group than the WOG group; however, the difference was not statistically significant (*p* = 0.043).

Table 2 shows the mean and standard deviation of the preparation time according to the instrument. The preparation time was significantly different for each group. The TRN group showed the least shaping time, followed by the WOG and PTG groups (*p* < 0.05).

**Table 2:** Means and standard deviations of canal preparation time (s)

Parameter	PTG	WOG	TRN
	Mean ± SD	Mean ± SD	Mean ± SD
Time a, b, c	245.75 ± 22.03	170.75 ± 24.72	139.10 ± 24.54

Statistically significant differences for total preparation time: a between PTG and WOG, b between WOG and TRN, c between PTG and TRN (*p* < 0.05)

## Discussion

Although NiTi files have continued to develop, it is still difficult to prepare S-shaped root canals. The aim of this study was to compare the canal transportation of PTG, WOG, and TRN in simulated S-shaped canals. As a result of the experiment, TRN showed significantly less transportation than the other groups at the 3 mm level from the apical curvature and 5 mm level from the coronal curvature (*p* < 0.05). Even though it was not significant at 1- and 4-mm levels, TRN had a lesser tendency to cause transportation than that seen in other groups. TRN also removed the least resin in the apical and coronal curvature sections (*p* < 0.05).

To compare the shaping ability of NiTi files, resin blocks were used instead of the extracted human teeth in this study. While human teeth have various anatomical morphology, simulated canals in resin blocks provide standardized conditions for study and allow for direct comparison of pre- and post-instrumentation images. However, there are some limitations that the hardness of resin blocks is different from the human teeth [16]. Consequently, instrumentation of resin blocks might not be similar to human teeth. In addition, this method cannot evaluate the cross section of root canal three-dimensionally. Nevertheless, numerous studies have used S-shaped simulated canals to compare the shaping ability of NiTi file systems [14, 17]. Furthermore, Khalilak et al. reported that the difference in apical transportation between extracted teeth and high-hardness resin blocks was similar [18]. Therefore, the results obtained from the resin blocks may be validated if carefully interpreted.

In all file systems, apical preparation size 25 was selected and a similar size of 26 was selected for TRN. In fact, the larger the apical preparation size, the more effective it is for debris removal and disinfection in the root canal [19]. However, in a previous study, Akhlaghi et al. reported that the ability of the 25 apical size to reduce bacteria was not significantly different from that of the other groups with greater apical sizes [20]. Furthermore, the more the apical size increases, the flexibility of the file decreases, so the risk of canal transportation also increases [21].

In this study, PTG was used in continuous sequence with S1, S2, F1, and F2 according to the manufacturer’s recommendations. A previous study showed similar anatomical outcomes between shaping a canal with ProTaper F2 using the single-file reciprocating technique and the conventional ProTaper full-sequence rotary approach [22]. However, the single-file F2 ProTaper technique displayed significantly lower debridement ability in oval-shaped canals compared with the conventional ProTaper full sequence [23]. In studies comparing the shaping ability of PTG with other single file systems, PTG has been used in the recommended sequence [24, 25].

TRN, the newly developed file system, is manufactured using a slim NiTi wire with a 0.8 mm maximum flute diameter and an off-centered parallelogram cross-sectional design, in addition to a special heat treatment. TRN exhibits higher flexibility and superior canal-centering ability while preserving tooth structure [11]. Recently, there have been several studies on the fatigue resistance of TRN. Riyahi et al. reported that TRN had greater cyclic fatigue resistance than ProTaper NEXT and Twisted files [15]. Elnaghy et al. also reported that TRN was more resistant to cyclic fatigue than the Vortex Blue and Race instruments in single and double curvature canals [12]. These findings, related to the enhanced fatigue resistance, could be attributed to the special heat treatment of the alloy and the design of the instruments. Heat treatment changes the transformation behavior of the alloy and thus increases the flexibility of NiTi endodontic instruments [26]. An off-centered parallelogram cross-sectional design and thin NiTi wire might have resulted in increasing cyclic fatigue resistance [11, 12, 27].

According to the experimental results, in the TRN group, significant deviations occurred only in the coronal regions at 6, 7, 8, and 9 mm (*p* < 0.05). On the other hand, the other groups showed significant deviations in the rest of the range except for 1 and 9 mm (*p* < 0.05). In addition, when comparing the degree of transportation of the three groups, the TRN caused less transportation in the apical section than that seen in the other groups and removed the least resin in the apical and coronal curvature. In other words, it can be said that the TRN had the best ability to maintain the center of canals in the apical curvature area.

The fact that the least amount of resin was removed in the TRN group might be related to a small and regressive taper and slim NiTi wire design. In the present study, the apical taper of PTG, WOG, and TRN was 0.08, 0.07, and 0.04, respectively. A significantly larger amount of resin was removed by PTG, compared to WOG, in the coronal curvature, and slightly higher in the apical curvature area. In addition, the PTG group showed more transportation than the other groups in the apical curvature (*p* < 0.05). This is consistent with a previous study showing that taper is a contributing factor in determining shaping ability [17, 28]. The greater the taper in the apical area, the lesser the flexibility and higher the transportation degree, compared to other files of the same size [29].

The preparation time is dependent on the number of instruments used, the operator experience, and on the technique used. In the present study, the preparation time included the time taken for active instrumentation, changing instruments, cleaning the flutes of instruments, and irrigation [25]. The TRN group showed the least preparation time followed by WOG and PTG. In general, using a single file system takes less time than using several sequential rotary files. Therefore, it might not be significantly important that the TRN and WOG had lesser time than the PTG. However, in two single file systems, the WOG and TRNshowed significant differences, presenting the better shaping efficiency of TRN than WOG.

## Conclusion

Under the conditions of this study, it can be concluded that TRN files maintained the original apical canal shape in double-curved simulated canals. In addition, TRN removed less resin than other heat-treated NiTi file systems. However, PTG with the largest apical taper showed more transportation in the apical curvature area.

## Data Availability

The datasets used and analyzed during the study are available from the corresponding author upon reasonable request.

## Abbreviations

NiTi: Nickel titanium
PTG: ProTaper GOLD
WOG: WaveOne GOLD
TRN: TruNatomy
